# Bovine dairy products and flow mediated dilation (FMD): a systematic review of the published evidence

**DOI:** 10.1007/s00394-024-03574-w

**Published:** 2025-01-24

**Authors:** Martina Rooney, Joyce Lambe, Aileen O’Connor, Simone Dunne, Andrea Mills, Emma L. Feeney, Eileen R. Gibney

**Affiliations:** 1 Food for Health Ireland, University College Dublin, Dublin 4, Republic of Ireland; 2 Institute of Food and Health, School of Agriculture and Food Sciences, University College Dublin, Dublin 4, Republic of Ireland

**Keywords:** Dairy, Flow mediated dilation, FMD, Endothelial function

## Abstract

**Purpose:**

Evidence suggests bovine dairy products may have neutral or beneficial effects on cardiometabolic health, despite being a source of saturated fat. The dairy matrix, the structure and combination of protein, fat, and other nutrients, and how they interact with each other, is purported to be responsible for these beneficial health effects. Whether this relationship extends to endothelial function, as assessed by flow mediated dilation (FMD), remains to be elucidated.

**Methods:**

Three electronic databases (PubMed, Embase and Cochrane Central) were searched from inception until 5th September 2024. This review included randomised controlled trials (RCT) investigating any bovine dairy intervention which considered endothelial function using FMD in humans with a non-dairy or alternative dairy control.

**Results:**

Of 4,220 records identified, 18 reports from 11 RCT including 508 (53.3% male) participants, examined endothelial function by FMD and were eligible for evidence synthesis. Eight papers reported an improvement, nine reported no effect and one reported a decrease in FMD. The greatest effects were found in those with impaired health at baseline, with whey protein and high dairy intakes observed to be most beneficial.

**Conclusion:**

Bovine dairy intake has neutral or beneficial effects on cardiometabolic health. This review demonstrates that this relationship extends to endothelial function as assessed by FMD. Whey protein and high dairy intakes may be most effective, although further high quality RCT in this area are warranted.

**Supplementary Information:**

The online version contains supplementary material available at 10.1007/s00394-024-03574-w.

## Introduction

Dairy products, such as milk and cheese, are an important dietary source of nutrients such as protein, fat, calcium, phosphorus, iodine, vitamin D, riboflavin and vitamin B12 and as such, contribute to many biological processes such as growth and development, bone health and efficient metabolism [1–3]. Nonetheless, dairy products also contain saturated fatty acids (SFAs) which have been negatively linked with cardiovascular disease (CVD) since the discovery of the ‘diet-heart hypothesis’ by Keys et al., in the 1980’s [4]. This has led dietary guidelines to recommend limiting SFA intakes to 10%, or less, of total energy intake [5–7]. However, more recent evidence has called these blanket recommendations into question, as not all foods containing saturated fat have the same negative cardiovascular effects and some are even postulated to be cardioprotective [8–11]. Calls for food-based translations of these nutritional guidelines have been made [12].

The ‘dairy matrix’, the complex structure of fats, proteins and other nutritional constituents and their interactions, has been suggested to be responsible for some of the variation (negative, neutral or beneficial effects) observed for dairy products on markers of metabolic health [9, 13–15], such as cholesterol concentrations, blood pressure (BP) [16, 17] and overall CVD risk and mortality [10].

Established markers of cardiovascular risk exist, including BP and lipid profiles, and are used routinely in examining the link between diet and health/disease. However, novel markers of vascular health have been developed in recent decades and are used in both research and tertiary care. One such example is, flow mediated dilation (FMD), a tool that is growing in its use as a risk marker within nutritional studies [18–20]. Endothelial function is essential for cardiovascular (CV) health and loss of endothelium-dependent vasodilation in the systemic arteries is known to occur in the pre-clinical phase of CVD [20]. The vascular endothelium, a single layer of endothelial cells lining the inside of blood vessels, controls vascular tone and blood flow through the synthesis and release of factors such as nitric oxide (NO) [21]. Earlier investigations of endothelial function involved catheterisation [22], however, technological advances now allow endothelial function to be assessed non-invasively through FMD. First introduced in 1992 [23], the technique requires occlusion of the brachial artery by a blood pressure cuff inflated to supra-systolic pressure for 5 min which induces reactive hyperaemia following release of the BP cuff [24–26]. Assessment of FMD is limited by need for expensive equipment and extensive training to obtain reproducible results, while consensus and evidence-based recommendations for FMD assessment was only published in 2019 [24]. FMD reference values for the evaluation of endothelial function and CV health have recently been published, with a value of 6.5% or more indicating optimal endothelial function [27]. Meta-analyses have identified that a 1% increase in FMD leads to a 12–13% reduced risk of CV event [28, 29], with some evidence that FMD is a stronger predictor of CV risk in diseased, rather than healthy, populations [30]. Furthermore, sex has been shown to alter the age-related decline in FMD, which may partly be explained through differences in baseline diameter [31]. While these published findings indicate a strong prognostic link between FMD and CV risk, analyses are hampered by high heterogeneity of included studies. Further non-invasive measures of vascular health exist, such as pulse wave velocity (PWV), and augmentation index (AIx), however these assess arterial stiffness and structure, rather than the functional properties of the endothelium [32].

Whilst a few systematic reviews and meta-analyses have considered the effect of dairy products on FMD, a paucity of published studies exists in this area. Ballard and Bruno [33] reported dairy foods benefit vascular function however they included any measure of vascular health, including PWV, AIx and FMD, making it difficult to draw concrete conclusion owing to the lack of comparability between outcome measures. Hajizadeh-Sharafabad et al., focused specifically on whey protein and found FMD increased by 9% after whey protein consumption, but had no effect on PWV or AIx [34]. A meta-analysis by Fewkes et al., found decreased FMD in response to a high-fat meal, although not all of the meals in the 90 included studies included dairy foods [35].

Dairy foods provide a wide range of nutrients important for health, and regular consumption is linked with improved cardiometabolic health, although the effect of dairy foods as a whole on FMD is not widely explored. Therefore, the aim of this review is to provide a comprehensive synthesis of randomised controlled trials (RCT) investigating the effect of any dairy food on FMD in adult cohorts. Furthermore, this work aims to examine the impact of study and population characteristics on the outcomes, to determine factors influencing variability.

## Methods

### Search strategy and study eligibility

The search strategy for a broad systematic review encompassing dairy and cardiometabolic health was developed and executed and is presented in the **Supplementary Material**. The present systematic review considers dairy and FMD only. The protocol for the review was registered with the PROSPERO database of systematic reviews (CRD42023486210) and follows Preferred Reporting Items for Systematic reviews and Meta-Analyses (PRISMA) guidelines [36]. A PICOS-based approach was used to design the search, where the population was human adults; the intervention was bovine dairy products; the comparator was no dairy, alternative dairy, alternative food or placebo; the outcome was FMD; and study type was RCT, and is presented in Table 1. No restriction was placed on the health status of the populations investigated. Studies were eligible for inclusion if they were RCT, published in full, and available in English. Studies were excluded if based on pregnant or lactating subjects or those on weight-reducing diets; fortified or enriched dairy products or those modified during manufacturing; probiotic-containing dairy; or dairy as a co-intervention (e.g. with exercise). Studies were included when the nutritional content of the test food was modified through alteration of the dairy herd feeding strategy, however studies were excluded if the nutritional content was altered by the addition of nutrients or ingredients during manufacture/processing. Yogurt was included as it naturally contains probiotics, however yogurt or other dairy foods with added probiotics were excluded. Time constraints and resources did not permit a full and systematic search for conference abstracts and therefore conference abstracts retrieved during the search were excluded from the analysis. The following databases were searched from inception to the 31st March 2023, and updated on 5th September 2024: PubMed, Embase and Cochrane Central Register of Controlled Trials.

**Table 1:** PICOS statement for the current systematic review

Descriptor	Inclusion criteria	Exclusion criteria
**P**articipants	Human adults of any health status	≤ 18 years of age Pregnant or lactating Animal or cell-line studies
**I**ntervention	Bovine dairy products	Non-bovine dairy intervention Co-interventions e.g., weight reducing diet
**C**omparator	No dairy, alternative dairy, alternative food or placebo	No comparator or control group
**O**utcome	Endothelial function as assessed by flow mediated dilation	Not flow mediated dilation
**S**tudy	Randomised controlled trial	Observational or non-randomised controlled trials

### Deduplication and screening

Deduplication was firstly carried out in EndNote reference management software package (Clarivate, London, UK), with additional automated and manual deduplication carried out in Covidence (Covidence systematic review software, Veritas Health Innovation, Melbourne, Australia) when the records were imported. Studies were screened at title and abstract level by two independent reviewers (JL 100%, AM 50%) with discrepancies resolved by discussion with a third reviewer (ERG). While screening all titles and abstracts in duplicate is the gold standard approach, this approach was taken given the lack of resources available and advice from the Cochrane Handbook for Systematic Reviews of Interventions [37]. Level of agreement was monitored throughout title and abstract screening, with disagreements of 14.8% after 12.5% of records were screened and 5% after 37.5%. All texts put forward for full text screening were reviewed by two independent reviewers (MR, JL), again with discrepancies resolved by discussion with a third reviewer (ERG), and in line with the Cochrane Handbook [37]. From the full set of studies retrieved for the broader systematic review, the subset of studies investigating the impact of dairy on FMD was identified and put forward for data extraction.

### Data extraction

A data extraction template was created in Covidence to collect study information and data were extracted in duplicate (JL, MR). Data were collected on trial design (parallel or crossover); duration; type, dose and timing of dairy; and %FMD response. Data on author, sponsorship, country, setting, cohort, inclusion and exclusion criteria, sample size, withdrawals/exclusions, and baseline characteristics were also extracted for quality assessment. Where necessary, data were extracted from published figures using Web Plot Digitizer 4.6 (Ankit Rohatgi, Pacifica, California, USA).

### Risk of bias

The risk of bias for each study was independently assessed by two reviewers (MR and JG) in Covidence using RoB 2: A revised Cochrane risk-of-bias tool for randomised trials [38] and any discrepancies were resolved by discussion. Bias was assessed over the following 7 domains; random sequence generation, allocation concealment, blinding of participants and personnel, blinding of outcome assessors, incomplete outcome data, selective reporting and other sources (including funding). Risk of bias was judged as Low, High or Unclear. A judgement on the overall risk of bias was reached based on the least favourable assessment across the domains of bias.

### Data synthesis and analysis

Summary characteristics of the eligible studies were presented in tabular form and a narrative synthesis of the respective FMD% results from the studies was created, stratifying by population type, BMI category, dairy type, mean age and study design. Owing to the high methodological heterogeneity of the eligible studies, meta-analysis was not possible in the present review.

## Results

**Fig. 1:**
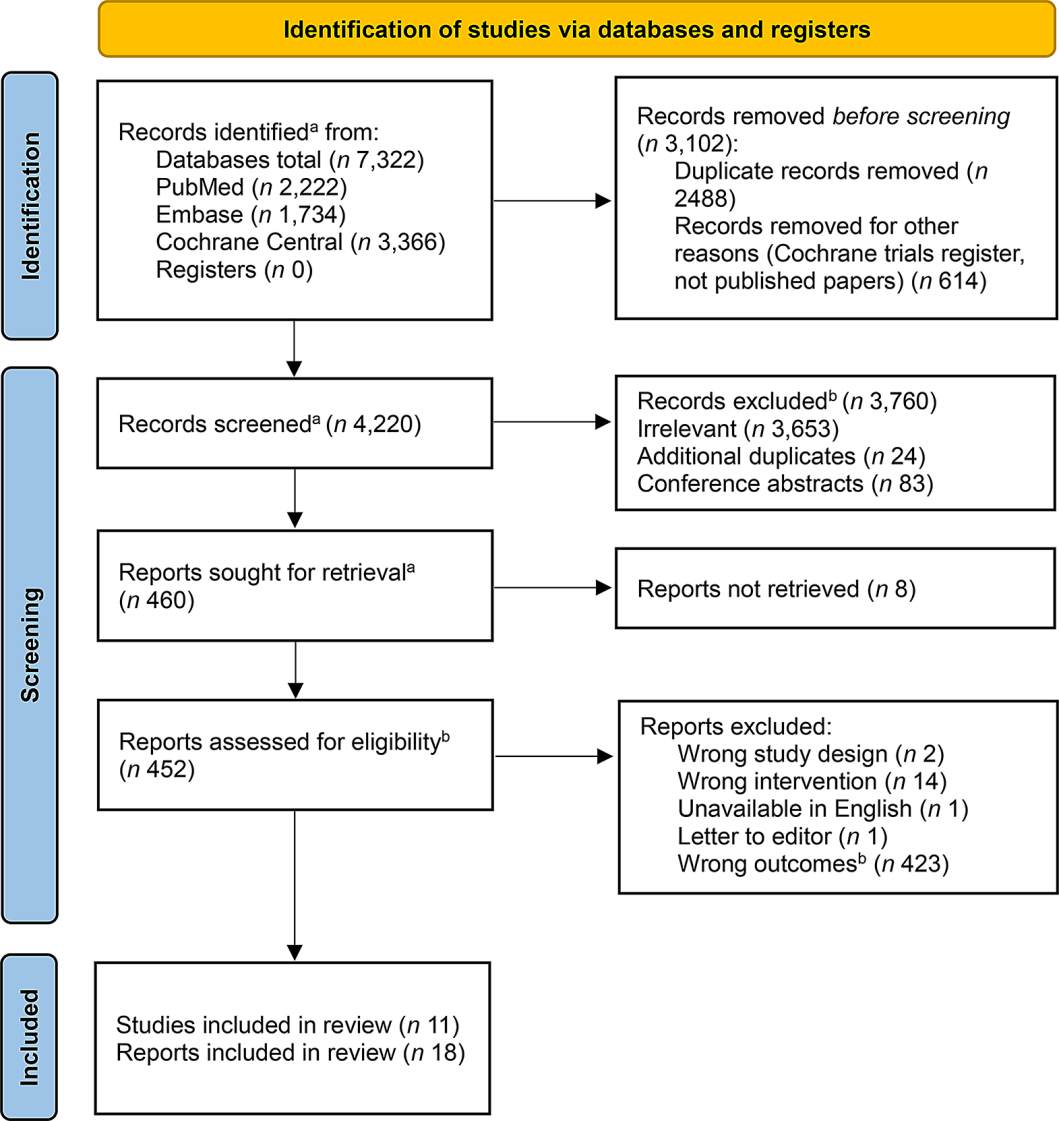
PRISMA flow diagram for the search strategy systematic review of the literature. **a** Records identified from full search for effect of dairy on intermediate biomarkers of cardiovascular disease (including blood lipids, blood pressure, glycaemic markers, inflammatory markers and FMD). **b** Eligibility for inclusion in systematic review of effect of dairy on FMD only

The PRISMA flow diagram illustrating the study selection process is presented in Fig. 1. For the broader systematic review search, a total of 7,322 records were retrieved from PubMed, Embase and Cochrane Central. In EndNote, 2,488 duplicates and 614 trial registrations were identified and removed. Of the 4,220 records imported into Covidence put forward for title and abstract screening, 3,760 were excluded on the basis of not meeting the inclusion criteria. At this stage, 460 records were retrieved, with access not possible for 8 records. The reports were assessed for eligibility for the effect of dairy on FMD and 11 studies were included in the final synthesis. Two studies [39, 40] reported from the same study and cohort and the paper with the most relevant data for the present review [40] was included for data extraction and synthesis.

### Characteristics of included studies

A summary of the participant characteristics included in the selected studies is presented in Table 2 and detailed study design information is provided in Table 3. Six of the studies were conducted in the USA [41–46], four in the UK [40, 48–49] and one in Switzerland [50]. All but one, a parallel RCT [50], had a crossover design. Six studies were acute and lasted 120 to 480 min [40–44, 49] and five were chronic studies lasting between 4 and 12 weeks in duration [45–48, 50]. Overall, the eligible RCTs included 508 participants, of which 271 (53.3%) were male and 237 (46.7%) were female. The sample sizes ranged from 19 [42] to 125 participants [50]. All studies investigated mixed sex samples with the proportion of females ranging from 11% [49] to 56% [50]. The mean age of the participants ranged from 26.1 years [43, 44] to 58 years [46]. The baseline BMI ranged from 25.0 kg/m^2^ [50]^50^] to 35.0 kg/m^2^ [42]. Three RCTs investigated healthy cohorts [43, 44, 50], and the remaining eight studies recruited those with an elevated CVD risk.

**Table 2:** Summary of study design and participant characteristics of 11 eligible studies

	*n* (%)
Geographic location	
’North America	6 (54.5)
’Europe (excluding UK)	1 (9.1)
’UK	5 (36.4)
Design	
’Parallel	1 (9.1)
’Crossover	10 (90.9)
Intervention duration	
’Post-prandial (< 12 h)	6 (54.5)
’≤ 4 weeks	3 (27.3)
’5–12 weeks	2 (18.2)
Sample size	
’< 50	7 (63.6)
’50–100	3 (27.3)
’> 100	1 (9.1)
Sex	
’Male	0 (0.0)
’Female	0 (0.0)
’Mixed	11 (100.0)
Age (y) mean	
’< 50	3 (27.3)
’≥ 50	8 (72.7)
BMI category (kg/m^2^)	
’< 25	0 (0.0)
’25-29.9	7 (63.6)
’> 30	4 (36.4)
Dairy foods investigated^a^	
’High dairy intakes	6 (33.3)
’Dairy products^b^	7 (38.9)
’Milk proteins	5 (27.8)
Health Status	
’Healthy	3 (27.3)
’Elevated blood pressure	4 (36.4)
’Increased CVD risk	4 (36.4)

^a^ Of 18 individual reports from 11 RCTs

^b^ Dairy products include low-fat milk, non-fat milk, butter, lactose and combined lactose and whey

Abbreviations: BMI, body mass index; CVD, cardiovascular disease; y, years

**Table 3:** Population and study design characteristics of the individual studies (*n* 11)

Author, Year, Country	Aim	RCT design	Duration		*n* (M/F)	Age, years	BMI, kg/m^2^	Participants	Dairy intervention(s), dose	Non-dairy intervention(s), dose
Ballard et al., 2013, USA [41]	To examine the effect of acute NOP-47 ingestion on FMD responses, biomarkers of vascular function.	Cross-over	Acute 120 min	21 (11/10)		55.2 ± 1.3	27.8 ± 0.6	Overweight, middle-aged, impaired FMD	Whey protein hydrolysate, (NOP-47), 5 g	Placebo (artificial sweetener), 5 g
Ballard et al., 2013, USA [42]	To examine postprandial FMD in response to low-fat milk or rice milk.	Cross-over	Acute 180 min	19 (14/5)		28.5 ± 2.2	35.0 ± 0.9	Obese subjects with metabolic syndrome	Low-fat milk (1%), 475 ml	Rice milk, 435 ml
Machin et al., 2015, USA [45]	To determine if conventional non-fat dairy products improve vascular function.	Cross-over	Chronic 4 weeks	49 (22/27)		53 ± 2	30.45	Elevated BP	High-dairy condition, 4 servings non-fat dairy/d	No dairy condition, 4 fruit products/d
Fekete et al., 2016, UK [48]	To test if milk protein supplementation improves vascular function.	Cross-over	Chronic 8 weeks	36 (20/16)		52.9 ± 2.1	27.1 ± 0.8	Pre-hypertension and mildly hypertensive	Whey-protein, 56 g/d Calcium caseinate, 56 g/d	Maltodextrin, 54 g/d
Radtke et al., 2017, Switzerland [50]	To show that a diet with butter with ruminant TFA is not inferior to a diet with margarine with/without TFA.	Parallel	Chronic 4 weeks	125 (55/70)		54.5 (48–61)	25 (22.5–28.4)	Healthy males and females	Alpine butter, rich in ruminant TFA, 33% of TEI	Margarine with 2% industrial TFA, or without TFA, 33% of TEI
Fekete et al., 2018, UK^a^ [49]	To determine if ingestion of milk proteins with ↑fat meals improves vascular function.	Cross-over	Acute 480 min	27 (24/3)		50.1 ± 2.3	27.3 ± 0.7	Mildly hypertensive	Whey protein, 28 g. Calcium caseinate, 28 g	Maltodextrin 27 g
Leary et al., 2018, USA^b^ [44]	To determine if non-fat milk attenuates postprandial hyperglycemia independent of its protein quantity.	Cross-over	Acute 120 min	29 (17/12)		26 ± 1	31.6 ± 0.9	Overweight and obese	Non-fat milk, 227 g Lactose, 12 g, whey, 8 g, water	n/a
Leary et al., 2018, USA^c^ [43]	To determine if non-fat milk attenuates postprandial hyper-triglyceridemia.	Cross-over	Acute 240 min	30 (18/12)		26 ± 1	31.5 ± 0.8	Obese subjects	Non-fat milk, 227 g Lactose, 12 g, whey, 8 g, water Lactose, 12 g, water	n/a
Roy et al., 2020, USA [46]	To determine the effects of whole milk and full-fat dairy products on vascular function.	Cross-over	Chronic 4 weeks	60 (28/32)		58 ± 2	29.3 ± 0.8	Elevated systolic BP	High dairy condition, whole milk, cheese, yogurt. 4 servings/d	No dairy condition, plant based products
Vasilopoulou et al., 2020, UK [47]	To investigate the impact SFA-reduced, MUFA-enriched dairy products, on CVD risk markers, compared conventional dairy products.	Cross-over	Chronic 12 weeks	50 (31/19)		52 ± 3	25.8 ± 0.5	Elevated CVD risk	Commercially available UHT milk, cheddar cheese, butter, ∼41 g/d dairy fat. ↑MUFA UHT milk, cheddar cheese, butter, ∼41 g/d dairy fat.	n/a
Markey et al., 2021^a^, UK [40]	To investigate the acute effect of sequential high fat mixed meals containing FA-modified dairy products on the postprandial %FMD response.	Cross-over	Acute 480 min	52 (31/21)		53 ± 2	25.9 ± 0.5	Those with a moderate risk of CVD	Commercially available UHT milk, 875 g; cheddar cheese, 47.6 g; butter, 48 g. ↑MUFA UHT milk, 777 g; cheddar cheese, 47.6 g; butter, 52.4 g.	n/a

Data presented as mean ± SD or median (IQR), unless otherwise stated

^a^ Testing protocol in Fekete et al. [49], and Markey et al. [40], comprised sequential meals of a breakfast at 0 min and lunch at 330 min

^b^ Testing protocol in Leary et al. [44], also included an oral glucose tolerance test (100 g glucose) at 0 min

^c^ Testing protocol in Leary et al. [43], also included a high-fat meal of corn chips (1 g dietary fat/kg body weight) at 0 min

Abbreviations: d, day, FMD, flow mediated dilation; OGTT, oral glucose tolerance test; TEI, total energy intake; TFA, trans fatty acid

### Intervention foods

Characteristics of study design are presented in Table 3. Non-fat dairy [43–45], low-fat milk [42], full-fat dairy [46] and butter [50] were investigated. Five RCTs investigated dairy proteins, including a proprietary whey protein hydrolysate, NOP-47 [41], whey protein and calcium caseinate in isolation [48, 49] and lactose was considered alone and in combination with whey protein [43, 44].

### FMD outcomes

The following section will stratify findings by population type, BMI category and age.

### Healthy

All FMD results are presented in Table 4. Considering the studies that examined a heathy population, one study recruited healthy, middle-aged participants with a BMI 20–30 kg/m^2^ and reported no significant differences in FMD after four weeks on intervention in response to a diet with alpine butter rich in ruminant trans fatty acids (TFA’s) compared to non-dairy products [50]. Sub-group testing within this study found alpine butter improved FMD% response in males, but not in females, although this analysis was not powered [50]. Two studies recruited young, apparently healthy adults with BMI ≥ 25 kg/m^2^. In one study, non-fat milk was found to attenuate the decrease in FMD in response to an oral glucose tolerance test (OGTT) in the cohort as a whole, and to increase in those with the highest central adiposity [44]. Combined lactose and whey were found to have no effect on FMD in response to an OGTT [44] and non-fat milk, combined lactose and whey, and lactose alone were found to have no effect on FMD in response to a high-fat meal [43].

**Table 4:** Main FMD% findings of the eligible studies (*n* 11)

Author, year	Dairy product	*n*	FMD%			Main findings
			Pre	Post	*P* value	
Ballard et al., 2013 [41]	Whey protein (NOP-47) Placebo	21 21	3.71 ± 0.51 4.46 ± 0.54	5.12 ± 0.51 4.30 ± 0.51	*P* < 0.005	Whey ↑ FMD% at 120 min compared to placebo
Ballard et al., 2013 [42]	Low-fat milk (1%) Rice milk	19 19	6.41 ± 0.95 6.17 ± 0.74	6.74 ± 1.03 5.98 ± 0.82	*P* > 0.05	No difference observed throughout time trial
Machin et al., 2015 [45]	High-dairy No dairy	47 47	3.01 ± 0.35 3.39 ± 0.33	4.09 ± 0.43 2.45 ± 0.43	*P* < 0.005	High-dairy ↑ FMD%
Fekete et al., 2016 [48]	Whey-protein, Ca caseinate Maltodextrin	36 36 36	4.79 ± 0.3 4.83 ± 0.3 4.79 ± 0.3	5.38 ± 0.4 4.94 ± 0.3 4.07 ± 0.3	*P* < 0.001	Whey and calcium caseinate ↑ FMD% compared to control
Radtke et al., 2017 [50]	Butter Margarine with TFA Margarine without TFA	50 31 48	5.0 (3.2–7.1) 4.7 (3.2–7.1) 4.3 (3.1–7.2)	5.5 (4.7–6.1) 5.2 (4.2–6.3) 6.3 (5.2–7.4)	0.987	No difference
Fekete et al., 2018 [49]	Whey-protein, Ca caseinate Maltodextrin	25 25 25	4.78 4.75 4.81	4.84 4.94 4.34	0.014	Whey and calcium caseinate ↑ FMD% compared to control
Leary et al., 2018 [44]	Non-fat milk Lactose, whey	29 29	7.6 ± 0.6 8.7 ± 0.6	8.4 ± 0.6 8.0 ± 0.7	NR	No difference in any of the trials
Leary et al., 2018 [43]	Non-fat milk Lactose and whey Lactose	30 30 30	9.0 ± 0.8 9.3 ± 0.8 7.8 ± 0.8	7.9 ± 0.l7 9.0 ± 0.9 9.1 ± 0.8	NR	No difference in any of the trials
Roy et al., 2020 [46]	High dairy No dairy	60 60	5.65 ± 0.54 6.44 ± 0.55	5.34 ± 0.62 5.57 ± 0.62	0.367	No difference
Vasilopoulou et al., 2020 [47]	Conventional dairy MUFA-enriched dairy	50 50	4.68 ± 0.28 4.42 ± 0.27	4.14 ± 0.30 4.77 ± 0.28	*P* < 0.0001	Compared to baseline, the conventional dairy diet decreased %FMD compared with an increase after ↑ MUFA dairy
Markey et al., 2021 [40]	Conventional dairy MUFA-enriched dairy	45 45	4.66 ± 0.28 4.50 ± 0.27	4.83 ± 0.34 5.20 ± 0.30	0.681	No treatment effect observed. Relative to baseline, there was a 4% higher ΔAUC for FMD% following MUFA-dairy compared to conventional dairy (*P* = 0.075).

Data presented as mean ± SEM, or median (interquartile range)

Abbreviations: ↑ increase; Ca, calcium; d, day; MUFA, monounsaturated fatty acid; NR, not reported

### Elevated BP

Four studies, comprising six reports, recruited individuals with elevated BP. FMD was found to increase in response to long-term consumption of non-fat dairy products [45] but no effect was observed after full-fat dairy [46]. Whey protein and calcium caseinate were found to increase FMD after 8 weeks compared to maltodextrin control [48] and to attenuate the decrease in FMD after a high-fat challenge [49] in the same cohort.

### Increased CVD risk

Four studies, comprising six reports, considered populations at increased risk of CVD. In a cohort of middle-aged adults with impaired FMD, ingestion of whey protein was found to increase FMD [41]. Low-fat milk was found to have no effect on FMD in those with metabolic syndrome [42]. In a cohort with an increased CVD risk based on a modified Framingham risk analysis, high intakes of MUFA-enriched dairy was found to increase FMD whereas high intakes of conventional dairy was found to decrease FMD after 12 weeks [47], with no effect on FMD in a post-prandial investigation of the same cohort [40].

### BMI 25.0–29.9 kg/m2

In seven studies, comprising eleven reports, participants with mean BMI ranging from 25.0 to 29.9 kg/m^2^ were recruited. Three of these studies investigated whey protein and observed an increase in FMD [41, 47] or attenuation of the decrease in FMD in response to a high-fat meal [49]. One study recruited volunteers with a mean BMI of 25 kg/m^2^, which found butter to have null effect on FMD, although a significant increase in FMD was observed in those with a BMI 25–30 kg/m^2^, however this analysis was underpowered [50]. There were inconsistent results in response to long-term high intakes of full-fat dairy products, with an increase [47], no effect [46] and decrease [47] in FMD reported, while a post-prandial study found FMD did not change in response to either MUFA-enriched or conventional dairy products [40].

### BMI ≥ 30 kg/m2

Four studies, comprising seven reports, recruited cohorts with a mean BMI ≥ 30 kg/m^2^ [42–45]. One study reported an increase in FMD in response to 4 weeks of non-fat dairy [46], there was one report of non-fat milk attenuating the decrease in FMD in response to an OGTT [44], while the remaining five reports observed no effect on FMD in response to non-fat milk [43], low-fat milk [42] or whey and/or lactose [43, 44]. However, in sub-group analysis non-fat milk was found to significantly increase FMD only in those with the highest tertile of android adiposity [44].

### Young cohorts

Three studies recruited cohorts with a mean age of 26-28.5 years, although two of these studies investigated the same cohort [43, 44]. All three cohorts were obese and all three studies were postprandial crossover trials [42–44]. One study investigated low-fat milk [42] while the other two considered non-fat milk, lactose, and lactose and whey in combination [43, 44]. Of six reports from these three studies, non-fat milk was found to attenuate the decrease in FMD in response to an OGTT, and the remaining investigations reported no effect.

### Middle-aged cohorts

The remaining eight studies all recruited subjects with a mean age between 50 and 58 years. There were five reports from three acute studies, where two studies reported an increase in FMD in response to whey and casein [41, 49], while MUFA-enriched dairy and conventional dairy were found to attenuate the decrease in FMD in response to a high-fat meal [40, 49]. There were seven reports from five long-term studies. Whey protein and calcium caseinate were found to increase FMD [47]. Butter had no effect in lean individuals but was found to increase FMD in those with a BMI 25–30 kg/m^2^ [50]. In response to high dairy intakes, MUFA-enriched [47] and non-fat dairy [45] were found to increase FMD, while full fat dairy was found to decrease FMD [47] or have no effect [46].

### The effect of food and study design on FMD outcomes

#### High dairy intake

High dairy intakes were classified as ≥ 3 servings per day, in line with European food based dietary guidelines where most European countries recommend 2–3 servings daily [51]. Six reports from four studies considered high dairy intakes in the present review [40, 45–47]. Chronic high intakes of non-fat dairy [45], and MUFA-enriched dairy foods [47] were found to increase FMD, while conventional full-fat dairy consumption had no effect [46] or decreased FMD [47]. All studies investigating high dairy intakes recruited middle-aged individuals who were at an increased risk of CVD.

#### Casein and whey protein

Two reports of casein [48, 49] and five reports of whey protein [41, 43, 44, 48, 49] on FMD are included in the present review. No studies considered whey and casein in combination. Whey and casein were found to increase FMD over 8-weeks and attenuate FMD response in both a chronic and a post-prandial investigation [48, 49]. In other acute trials, whey protein was found to increase FMD at 30 min and 120 min compared to baseline [41] while combination of whey with lactose was found to have null effect [43, 44]. In the studies where milk proteins were found to improve FMD, middle-aged cohorts at increased risk of CVD were investigated [41, 48, 49], whereas in the studies which reported no effect, younger, overweight and obese, apparently healthy individuals were recruited [43, 44].

#### Individual dairy products

Acute intakes of low-fat milk [42] and lactose [43] and chronic consumption of butter [50] were all found to have no effect on FMD. Non-fat milk was found to attenuate the decrease in FMD in response to an OGTT in one study [44], but not in response to a high-fat challenge in another [43]. Despite these findings, body composition may be an important factor, as butter was found to increase FMD in those with a BMI of 25–30 kg/m^2^, [50] and non-fat milk was found to significantly increase FMD in those in the highest tertile of android adiposity [44], results which were not observed in leaner individuals.

#### Liquid vs. semi-solid dairy

The effect of different forms of dairy on endothelial function is worth considering. Liquid dairy was tested in six trials [41–44, 48, 49], one study investigated semi-solid dairy [50], while four considered a variety of dairy forms [40, 46–47]. Low-fat [42] and non-fat milk [43, 44] were found to have no effect on FMD, while whey and/or casein in solution with water were shown to improve FMD in three studies [41, 48, 49], but not when the test protocol involved a glucose or fat challenge [43, 44]. Butter was the only semi-solid dairy food investigated, however, no effect on endothelial function was observed [50]. Results were inconsistent from the studies where mixed forms of dairy, i.e., milk, cheese and butter, were tested simultaneously [40, 45–48].

#### Challenge tests

Four post-prandial studies, comprising eight reports, included high-fat or glucose challenge tests [40, 43, 44, 49]. Non-fat milk was found to attenuate the decrease in FMD in response to an OGTT, while a combination of whey and lactose had no effect [44]. Non-fat milk, whey and lactose in combination, and lactose were found to have no effect on FMD in response to a high-fat, non-dairy meal [43]. Fekete et al. [49] and Markey et al. [40], both investigated the FMD in response to a high-fat dairy meal. Both studies employed sequential high fat, high-dairy meals into their trials, and while Markey et al., investigated the effect of MUFA-enriched vs. conventional milk, butter and cheese, Fekete et al., considered the effect of whey protein and calcium caseinate on the FMD response to a high-fat dairy challenge. Fekete et al., found whey protein attenuated the expected decrease in FMD in response to a high-fat dairy challenge [49]. Markey et al., found conventional dairy to have no effect on FMD response after sequential high fat meals whereas a 4% higher response for FMD after the MUFA-enriched, compared to conventional, dairy was found although this was not significant (*P* = 0.075) [40].

#### Acute vs. chronic

Two acute trials did not incorporate a challenge test, with one reporting an increase in FMD in response to whey protein compared to baseline [41], and the other reporting no effect in response to low-fat milk [42]. Five trials, comprising seven reports, conducted chronic investigations of 4–12 weeks duration [45–48, 50]. Whey and casein were found to increase FMD [48] and butter was found to have no effect [50]. There were four investigations into high-dairy intakes, with conflicting results. Non-fat dairy and MUFA-enriched dairy were found to increase FMD [45, 47], while full-fat dairy was found to decrease FMD [47] or have no effect [46].

### Clinical relevance

Of the studies where FMD increased in response to dairy intervention, three can be classified as being clinically meaningful [41, 45, 48], based on meta-analyses indicating a 8–13% lower risk of CV event per percent point increase in FMD [28–30]. A chronic study by Fekete et al., found whey and casein to increase FMD by 1.3% and 0.9%, respectively, compared to control [48], while less marked responses of 0.5% and 0.6%, respectively, were observed in the postprandial investigation [49], in those with elevated BP. A post prandial study by Ballard et al., found whey to increase FMD by 1.4% compared to placebo in those with impaired FMD [41]. In studies investigating high intakes of mixed dairy, Machin et al., found 4 servings per day of non-fat dairy to increase FMD by 1% compared to baseline and 1.6% compared to a non-dairy control a hypertensive cohort [45], whereas Vasilopoulou et al., reported a 0.36% increase in FMD in response to MUFA-enriched dairy, a 0.63% increase compared to conventional dairy [47], although it must be noted the only difference between interventions here was the fatty acid content of the intervention foods.

### Risk of bias

**Fig. 2:**
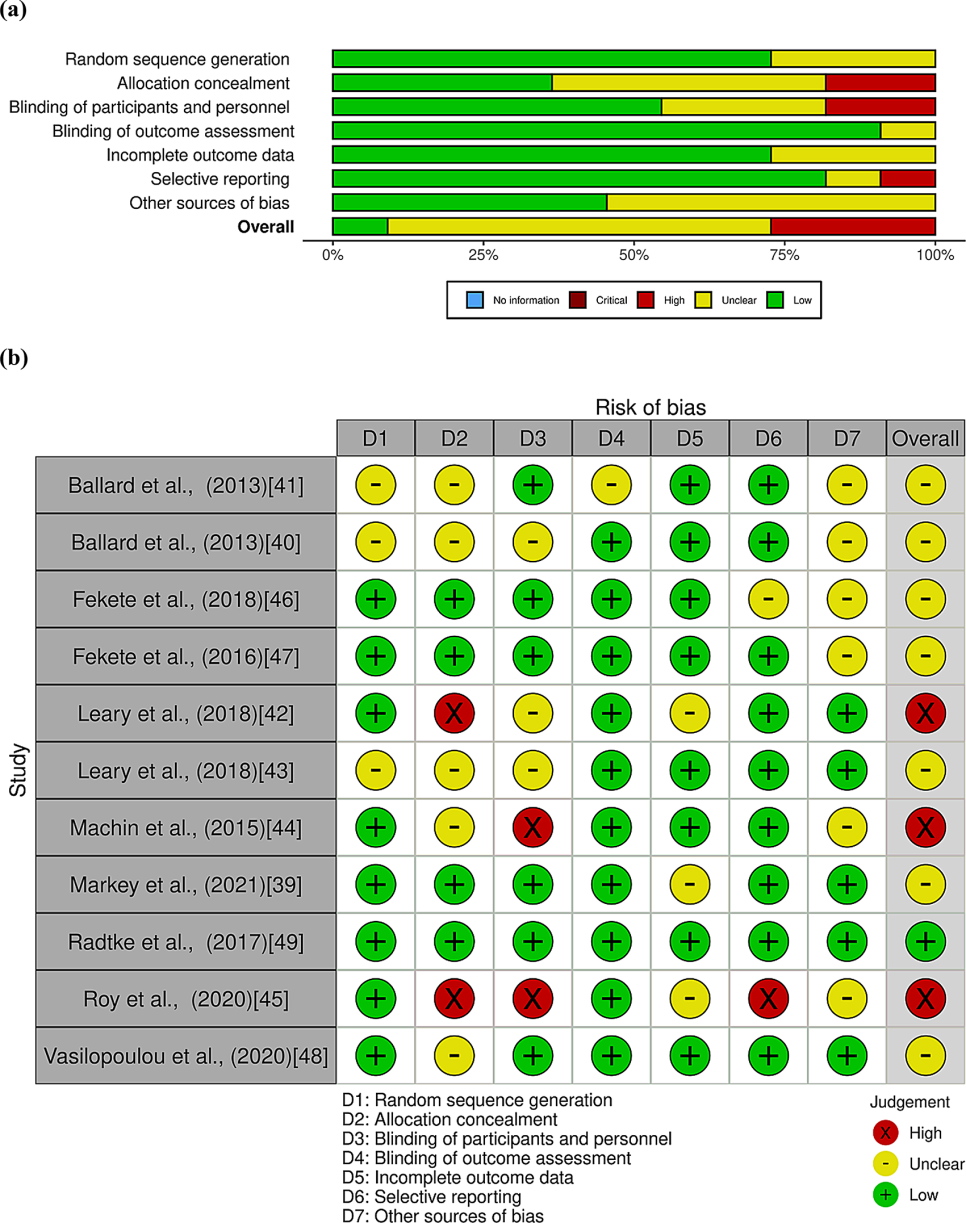
Risk of bias graph (**a**) and summary (**b**) of the included studies using the RoB2 tool in Covidence [38]. Level of risk is indicated as: +, low risk; -, low risk; X, high risk

Risk of bias assessments are presented in Fig. 2**(a) and (b)**. Across all outcome domains, only one study was judged to have an overall low risk of bias [50], with seven studies having an overall unclear risk of bias [33, 40–43, 47] and three studies having an overall high risk of bias [44–46]. Lack of clarity or detail about allocation concealment, blinding of participants and the role of dairy industry funders were the main reasons for judgments of unclear or high risk of bias.

## Discussion

This systematic review aimed to investigate the role of dairy products on endothelial function, as assessed by FMD. Of eighteen investigations from eleven eligible RCT, there were eight records reporting dairy foods improving FMD, nine finding no effect and one reporting a decrease in FMD. Whilst overall, these results are somewhat varied, they are in line with the totality of existing evidence, indicating dairy foods have a neutral or beneficial effect on cardiometabolic health [8, 11, 52, 53]. To the best of our knowledge this is the first systematic review to synthesise the evidence of the effect of dairy foods on endothelial function, as assessed by FMD, although, a thorough narrative review of the protective role of dairy on vascular function is presented elsewhere [33].

Observational evidence has demonstrated a linear relationship between dairy intakes and vascular health [17, 54]. In the current review, four RCT investigated the impact of high-dairy intakes on FMD [40, 45–47]. Within these studies, clinically relevant improvements in fasting FMD in response to non-fat dairy [45] and MUFA-enriched dairy [47] were observed, although such improvements were not seen in response to full-fat dairy [46, 47], with no change in FMD in response to a high-dairy fat meal in the post-prandial study [40]. These studies, from two centres in the UK and USA, recruited middle-aged individuals with impaired CV health; either elevated BP [45, 46] or an increased risk of CVD based on a modified Framingham Risk Score [40, 47], although BMI varied between the studies, with those in the American studies having a BMI of ≈ 30 kg/m^2^ [45, 46], while those in the UK studies were leaner, with a BMI ≈ 26 kg/m^2^ [40, 47]. Despite conflicting results from these studies, they are in line with existing evidence where dairy foods, with the exception of butter, have been found to have neutral or beneficial effects on cardiometabolic health [8, 11, 52, 53]. This review adds to this evidence base, identifying a similar relationship for FMD. One anomaly, however, is the decrease in FMD in response to conventional, full-fat dairy found by Vasilopoulou et al. [47],, although perhaps the inclusion of butter in the study protocol may offer some explanation, as the benefits of dairy products on cardiometabolic health have not been demonstrated for butter [55], perhaps given the high fat and low protein content, thus limiting the dairy matrix effect. The studies by Machin, Roy and colleagues incorporated milk, cheese and yogurt, whereas the RCTs by Vasilopoulou and Markey et al., added milk, cheese and butter into the dietary regime of their participants. Replacement of SFA with unsaturated fats have been shown to reduce risk of CV event and mortality [5, 7], which may explain the improved endothelial function observed in response to MUFA-enriched dairy, although other published evidence on MUFA and FMD is inconclusive [56, 57]. Furthermore, dairy contains many bioactive compounds which play a role in vascular function [58, 59]. The amino acid profile of dairy and whey, specifically arginine, is worth noting. Several RCT have pointed to a potential role of arginine in altering endothelial function and improving FMD, particularly in those with low arginine status [60–62] thus highlighting the importance of arginine in maintaining normal endothelial function. Nonetheless, these findings further support the role of the dairy matrix, the complex structure of protein, fat, and other nutritive compounds and their interactions in dairy foods, in cardiometabolic health.

The most convincing evidence from the present review comes from independent intervention with whey or casein. Dairy proteins were found to improve FMD in three RCT [41, 48, 49], while no effect was observed when whey was combined with lactose in another two studies [43, 44]. Moreover, in two of these studies, the improvements in FMD (> 1%) were clinically meaningful, equating to > 8% reduced risk of future CVD event [28–30]. A recent systematic review and meta-analysis reported whey protein consumption increased FMD, but had no effect on other measures of vascular health including AIx or PWV, although similar to the present review, there was high heterogeneity between the studies [34]. Hajizadeh-Sharafabad et al., included 153 subjects from six trials in pooled analysis for FMD, and sub-group analysis found no detectable effect of whey protein on FMD in healthy individuals or those aged < 45 years, while a significant increase in FMD was demonstrated in those at increased risk of CVD [34]. This is in agreement with the findings of the current review where the studies reporting an effect, were conducted in middle-aged cohorts at increased risk of CVD, while the studies by Leary and colleagues, with no effect, were conducted in younger, apparently healthy individuals [43, 44]. Whey may mediate vascular health through inhibition of angiotensin-I converting enzyme (ACE), inhibition of release of endothelin-1, stimulation of bradykinin activity or enhanced NO production [63–65]. Whey is made up of several proteins including β-lactoglobulin and immunoglobulins which have a range of properties which may contribute to the vasodilatory effects of whey protein [59]. A limitation of the present review is that whey and casein were not specifically included in the search strategy, as the focus was on dairy foods, not components of, thus some relevant papers may not have been identified. Nevertheless, despite the limited number of papers included here the findings are in agreement with other published systematic reviews and meta-analyses, where whey and casein are beneficial for vascular health, although middle-aged, at-risk populations may benefit more than others [34, 63, 66].

Impaired vascular health at baseline may offer some insight into the findings observed in the present review, as the largest impacts was observed in cohorts with impaired vascular health at baseline [41, 45, 47, 48]. Calls have been made for increased research into the long-term effects of dairy products, such as whey protein, on FMD response specifically in those with vascular dysfunction to fully elucidate this relationship [34]. In the present review, dairy intakes were found to increase [45, 48] or attenuate [49] FMD in cohorts with elevated BP at baseline, in all but one study [46]. A similar trend was observed for those at increased CVD risk, with dairy consumption found to increase FMD in most [41, 47], but not all investigations [40, 47]. Previous meta-analyses have shown FMD to be more strongly related to future CVD risk in diseased, rather than healthy, populations [28, 30], thus the results observed here suggest dairy products may be an effective dietary approach to manage endothelial function in those at increased CVD risk. Indeed, a 1% increase in FMD is associated with 10–13% reduced risk of future CV event [30] therefore the results from some of these studies may be of clinical relevance.

Evidence has linked increased consumption of saturated fat with elevated concentrations of LDL cholesterol, which is an established risk factor for CVD [67]. Therefore, despite contributing a range of nutrients important for health [68], food-based dietary guidelines often recommend limiting the intake of dairy products [5, 7]. More recently, these guidelines have been called into question, as meta-analyses have reported neutral or beneficial effects of dairy foods on cholesterol concentrations [8, 11], BP [69] and CVD outcomes [52, 53], with the most favourable effects observed for low-fat dairy and fermented dairy, including cheese. The dairy matrix has been thought to contribute to the cardioprotective nature of dairy products [9, 70]. The present review further adds to the evidence base and offers new insight into the role of dairy products and endothelial function as assessed by FMD. Furthermore, as observed in some of the studies included here [40, 47], modifying dairy foods by altering the dairy cow feeding strategies is an attractive and feasible strategy for creating dairy products with additional functionality. Such modification also allows further investigation into the role of protein, fat and other nutrients in the dairy matrix, which may help further our understanding. Combined with the cardioprotective effects of dairy products, further innovation in this area may lead to the development of dairy products with added health benefits while also maintaining the important health effects of the dairy matrix structure.

Assessment of risk of bias is regarded as an essential component of a systematic review on the effects of an intervention [38] as bias arising from flaws in the design and conduct of the study, can lead to an overestimation or underestimation of the true intervention effect [36]. Of the eleven included studies, only one was deemed to have a low risk of bias across all domains [50]. Therefore, the potential influence of bias on the interpretation of results needs to be considered. While eight of the studies [40, 43, 45–50] provided sufficient evidence of random sequence generation, only four provided sufficient detail about allocation concealment for the studies to be considered at low risk for selection bias. Evidence suggests intervention effect estimates tend to be exaggerated by 10% in trials with inadequate/unclear (versus adequate) allocation concealment [36]. Ten of the eleven studies in the review were crossover studies, and thus the risk of bias from carry over effects needs to be considered. Of the ten studies, six were postprandial, with washout periods ranging from at least one week [41, 43, 44] to eight weeks [40]. Researchers in these studies appeared to consider the wash-out period carefully, with consideration of the effect of the menstrual cycle on FMD in females demonstrated [42, 49], although only one study reported testing for carryover effects [46]. Furthermore, it should be noted the results observed in the present analysis may be associated with changes in LDL cholesterol concentrations, however this was not included in the synthesis. Half of the studies in this review were funded, at least in part, by dairy-related industries or organisations [42–47]. An updated Cochrane review found industry sponsored studies were 34% more likely to report favourable overall conclusions compared to non-industry funded studies [71]. Only two of the six dairy industry-funded studies in this review stated that the sponsors had no role in the design of study; collection, analyses or interpretation of data; writing of manuscript; or decision to publish [43, 44] and therefore, results should be interpreted with caution.

To the best of the author’s knowledge, this is the first systematic review to explore the relationship between FMD and the broad spectrum of dairy foods. Inclusion of RCT investigating all types of dairy allows comparison between food types and is a strength of the present work, although the term ‘dairy food’ is a blanket term and encompasses all foods under this umbrella term, including those not mentioned within the current analysis, The review followed a robust systematic approach [72] with data extraction completed in duplicate by the same two researchers. Nonetheless, this work is not without limitations. As studies containing any dairy foods were included and only small number of trials reported FMD there was high heterogeneity between the studies owing to clinical and methodological diversity, thus it was not suitable to conduct a meta-analysis [73]. Whey and casein were not included in the search strategy and therefore this review omits some relevant texts. Owing to the small number of studies, the authors were unable to elucidate if any sex-dependent effects were present. Furthermore, as no eligible study contained fermented dairy only, the analysis is unable to draw comparisons between fermented and non-fermented dairy foods, thus can be noted as a gap in the literature. Nonetheless, our findings are in line with the published evidence syntheses, however future reviews in this area should include whey and casein as search terms. Finally, this evidence synthesis is a secondary analysis of the main research question, as stated in PROSPERO.

## Conclusion

The evidence provided indicates that dairy foods have a predominantly neutral or potentially a beneficial effect on FMD. However, the reported positive effects were associated with specific dairy components, such as whey protein, or low-fat and MUFA-enriched dairy. Of eleven eligible studies comprising eighteen reports on dairy foods and FMD, eight reported an improvement in FMD, nine reported no effect, and one reported a decrease in FMD. These findings align with the broader evidence indicating neutral or beneficial effects of dairy foods on other cardiometabolic markers. Despite high methodological heterogeneity between studies and some concerns regarding bias, particularly in allocation concealment and the role of funders in study design and communication, further robust RCTs are warranted to clarify the relationship between specific dairy types and FMD. Overall, this review finds that dairy foods can be part of a healthy, balanced diet without adverse effects on cardiovascular health.

## Electronic supplementary material

Below is the link to the electronic supplementary material.

Supplementary Material 1

## Declarations

### Research transparency and reproducibility

The individual search strategy for each database searched is provided as supplementary material and can be made publicly available.
