# Supplementary material for: Bovine dairy products and flow mediated dilation (FMD): a systematic review of the published evidence
Source: Eur J Nutr. 2025 Jan 24;64(2):66. doi: 10.1007/s00394-024-03574-w (PMC11761514; doi:10.1007/s00394-024-03574-w)
Supplement: Supplementary file 1 — Supplementary Material 1 [file 394_2024_3574_MOESM1_ESM.docx]

**Title: Dairy products and flow mediated dilation (FMD): A systematic review of the published evidence**

Martina Rooney^1,2^, Joyce Lambe^1,2^, Aileen O’Connor^1,2^, Simone Dunne^1,2^, Andrea Mills^1,2^, Emma L Feeney^1,2^, Eileen R Gibney^1,2*^.

^1^ Food for Health Ireland, University College Dublin, Dublin 4, Republic of Ireland.

^2^ Institute of Food and Health, School of Agriculture and Food Sciences, University College Dublin, Dublin 4, Republic of Ireland.

*Corresponding author: Eileen R. Gibney, Tel.: +353-1-716-2819 Email: eileen.gibney@ucd.ie

**Supplementary Material**

**Supplemental table 1.** Search strategy for systematic review of genotypic and phenotypic factors affecting response to dairy on intermediate biomarkers of cardiovascular disease

| **Concept** | **Limiters** |
| --- | --- |
| **#1 Intake of dairy products** | All dairy products including milk or cheese or yogurt or fermented dairy products or cultured dairy products or buttermilk or milk powder or ice cream or dairy fat or curd or kefir or butter or dairy cream or milk protein isolates  AND  intake or consumption or servings |
| **#2 Genetic factors** | Genetics or single nucleotide polymorphisms or genotypes or genetic variation or alleles or genetic risk scores or diet-gene interactions or nutrigenetics or nutrigenomics or gene-nutrient interactions or Genome Wide Association Studies or genes or lipidomics |
| **#3 Phenotypic factors** | Cardiometabolic risk factors or inflammatory factors or body weight or body mass or age or sex or ethnicity or race or baseline health status (healthy, cardiovascular disease, diabetes) or pre- or post-menopause |
| **#4 Cardiometabolic outcomes** | Blood lipid levels or lipoprotein levels or blood pressure or blood glucose levels or insulin levels or insulin resistance or biomarkers of inflammation or metabolic syndrome or results of flow-mediated dilation or endothelial dysfunction or body weight or body mass or adiposity |
| **#5 Study type** | Randomised controlled trials |
| **#6 Study population** | Human, adult population groups |

**Supplemental table 2.** Syntax for searching PubMed**.**

| **Concept** | **Search** | **# records retrieved** |
| --- | --- | --- |
| #1 Dairy intake | ("Dairy Products"[Mesh] OR Dairy[tiab] OR “dairy product”[tiab] OR “dairy products”[tiab] OR milk[tiab] OR cheese[tiab] OR yogurt[tiab] OR yoghurt[tiab] OR “fermented dairy”[tiab] OR “cultured dairy”[tiab] OR buttermilk[tiab] OR “milk powder”[tiab] OR “ice cream”[tiab] OR “cultured milk product*”[tiab] OR “dairy fat”[tiab] OR curd[tiab] OR kefir[tiab] OR butter[tiab] OR cream[tiab]) AND (intake[tiab] OR consumption[tiab] OR consume*[tiab] OR serving*[tiab]) | 47,795 |
| #2 Genetic factors | "Genetic Association Studies"[Mesh] OR "Lipoproteins/genetics"[Mesh] OR "Diet/genetics"[Mesh] OR "Polymorphism, Single Nucleotide"[Mesh] OR Genotype[tiab] OR genetic*[tiab] OR “genetic variation”[tiab] OR allel*[tiab] OR “single nucleotide polymorphism*”[tiab] OR SNP[tiab] OR SNPs[tiab] OR “lipid polymorphism*”[tiab] OR “genetic risk score”[tiab] OR GRS[tiab] OR “diet-gene interaction”[tiab] OR “genetic heterogeneity”[tiab] OR “genetic varia*”[tiab] OR nutrigenetics[tiab] OR nutrigenomics[tiab] OR “gene-nutrient interaction”[tiab] OR GWAS[tiab] OR GeNuIne[tiab] OR gene[tiab] OR genes[tiab] OR lipidom*[tiab] | 3,658,282 |
| #3 Phenotypic factors | "Cardiometabolic Risk Factors"[Mesh] OR "Inflammation/metabolism"[Mesh] OR Phenotyp*[tiab] OR “body weight”[tiab] OR “body mass index”[tiab] OR BMI[tiab] OR obesity[tiab] OR obese[tiab] OR overweight[tiab] OR “waist circumference”[tiab] OR age[tiab] OR sex[tiab] OR ethnic*[tiab] OR race[tiab] OR dyslipidemia[tiab] OR “cardiovascular disease”[tiab] OR “heart disease”[tiab] OR CHD[tiab] OR CVD[tiab] OR diabetes[tiab] OR T2D[tiab] OR T1D[tiab] OR stroke[tiab] OR hypertension[tiab] OR “health status”[tiab] OR healthy[tiab] OR premenopausal[tiab] OR post-menopausal[tiab] OR postmenopausal[tiab] | 6,547,037 |
| #4 Cardiometabolic outcomes | "Dyslipidemias"[Mesh] OR "Biomarkers/blood"[Mesh]  OR Cholesterol[tiab] OR TC[tiab] OR LDL[tiab] OR HDL[tiab] OR VLDL[tiab] OR triglyceride*[tiab] OR triacylglycerol*[tiab] OR TG[tiab] OR dyslipidemia*[tiab] OR hyperlipidemia[tiab] OR hypertriglyceridemia[tiab] OR lipids[tiab] OR “blood lipids”[tiab] OR lipoprotein*[tiab] OR apolipoprotein*[tiab] OR apoa[tiab] OR apob[tiab] OR apoc[tiab] OR apod[tiab] OR apoe[tiab] OR apoh[tiab] OR “blood pressure”[tiab] OR systolic[tiab] OR hyperten*[tiab] OR “arterial pressure”[tiab] OR “pulse pressure”[tiab] OR “blood glucose”[tiab] OR “plasma glucose”[tiab] OR “glycated haemoglobin”[tiab] OR “glycated hemoglobin”[tiab] OR “glycosylated haemoglobin”[tiab] OR “glycosylated hemoglobin”[tiab] OR glycemi*[tiab] OR HBA1c[tiab] OR QUICKI[tiab] OR insulin[tiab] OR “homeostatic model assessment”[tiab] OR HOMA*[tiab] OR “insulin resistance”[tiab] OR “c-reactive protein”[tiab] OR “interleukin 6”[tiab] OR IL6[tiab] OR “metabolic syndrome”[tiab] OR “syndrome X”[tiab] OR “flow-mediated dilation”[tiab] OR “flow-mediated vasodil*”[tiab] OR “endothelial function”[tiab] OR “endothelial dysfunction”[tiab] OR “body weight”[tiab] OR “weight gain”[tiab] OR waist[tiab] OR “body mass index”[tiab] OR BMI[tiab] OR adiposity[tiab] OR obesity[tiab] | 2,947,445 |
| #5 RCTs | "Controlled Clinical Trial" [Publication Type] OR **“**Randomized controlled trial”[tiab] OR “randomised controlled trial”[tiab] OR “randomized intervention” OR “randomised intervention”[tiab] OR randomly[tiab] OR randomi*[tiab] OR RCT[tiab] OR “controlled clinical trial”[tiab] OR “Mendelian randomi*”[tiab] | 1,454,606 |
| #6 Human studies | "Humans"[Mesh] OR volunteer*[tiab] OR participant*[tiab] OR patient*[tiab] OR subject*[tiab] OR men[tiab] OR women[tiab] OR individual[tiab] OR individuals[tiab] OR adult*[tiab] OR “ethnic group*”[tiab] | 24,970,532 |
|  | #1 AND (#2 OR #3) AND #4 AND #5 AND #6 | 2,332 |

**Supplemental Table 3.** Syntax for searching Embase.

| **Concept** | **Search** | **No. of records retrieved** |
| --- | --- | --- |
| #1 Dairy intake | ('dairy product'/exp OR 'dairy product' OR 'milk'/exp OR milk OR 'cheese'/exp OR cheese OR 'yoghurt'/exp OR yoghurt OR 'fermented dairy product'/exp OR 'fermented dairy product' OR 'buttermilk'/exp OR buttermilk OR 'milk powder'/exp OR 'milk powder' OR 'ice cream'/exp OR 'ice cream' OR 'curd'/exp OR curd OR 'kefir'/exp OR kefir OR 'butter'/exp OR butter OR 'dairy cream'/exp OR 'dairy cream') AND (intake OR 'consumption'/exp OR consumption OR consume* OR servings) | 70,144 |
| #2 Genetic factors | 'genetic association study'/exp OR 'genetic association study' OR 'human genetics'/exp OR 'human genetics' OR 'single nucleotide polymorphism'/exp OR 'single nucleotide polymorphism' OR 'genotype'/exp OR genotype OR 'genetic variability'/exp OR 'genetic variability' OR 'genetic heterogeneity'/exp OR 'genetic heterogeneity' OR 'allele'/exp OR allele OR 'lipid polymorphism' OR 'genetic risk score'/exp OR 'genetic risk score' OR 'gene diet interaction'/exp OR 'gene diet interaction' OR 'nutrigenetics'/exp OR nutrigenetics OR 'nutrigenomics'/exp OR nutrigenomics OR 'gene-nutrient interaction' OR 'genome-wide association study'/exp OR 'genome-wide association study' OR 'gene'/exp OR gene OR 'lipidomics'/exp OR lipidomics OR 'lipidome'/exp OR lipidome | 5,308,674 |
| #3 Phenotypic factors | 'cardiometabolic risk factor'/exp OR 'cardiometabolic risk factor' OR 'inflammation'/exp OR inflammation OR 'phenotype'/exp OR phenotype OR 'body weight'/exp OR 'body weight' OR 'body mass'/exp OR 'body mass' OR 'obesity'/exp OR obesity OR 'obese patient'/exp OR 'obese patient' OR 'waist circumference'/exp OR 'waist circumference' OR 'age'/exp OR age OR 'gender and sex'/exp OR 'gender and sex' OR 'ethnic group'/exp OR 'ethnic group' OR 'race'/exp OR race OR 'dyslipidemia'/exp OR dyslipidemia OR 'cardiovascular disease'/exp OR 'cardiovascular disease' OR 'heart disease'/exp OR 'heart disease' OR 'ischemic heart disease'/exp OR 'ischemic heart disease' OR 'diabetes mellitus'/exp OR 'diabetes mellitus' OR 'cerebrovascular accident'/exp OR 'cerebrovascular accident' OR 'hypertension'/exp OR hypertension OR 'health status'/exp OR 'health status' OR 'normal human'/exp OR 'normal human' OR 'premenopause'/exp OR premenopause OR 'postmenopause'/exp OR postmenopause | 16,688,665 |
| #4 Cardiometabolic outcomes | 'dyslipidemia'/exp OR dyslipidemia OR 'biological marker'/exp OR 'biological marker' OR 'cholesterol ester'/exp OR 'cholesterol ester' OR 'triacylglycerol'/exp OR triacylglycerol OR 'hyperlipidemia'/exp OR hyperlipidemia OR 'hypertriglyceridemia'/exp OR hypertriglyceridemia OR 'lipid'/exp OR lipid OR 'lipid blood level'/exp OR 'lipid blood level' OR 'lipoprotein'/exp OR lipoprotein OR 'apolipoprotein'/exp OR apolipoprotein OR 'apolipoprotein a'/exp OR 'apolipoprotein a' OR 'apolipoprotein b'/exp OR 'apolipoprotein b' OR 'apolipoprotein e'/exp OR 'apolipoprotein e' OR 'apolipoprotein c'/exp OR 'apolipoprotein c' OR 'apolipoprotein d'/exp OR 'apolipoprotein d' OR 'apolipoprotein h'/exp OR 'apolipoprotein h' OR 'blood pressure'/exp OR 'blood pressure' OR 'systolic blood pressure'/exp OR 'systolic blood pressure' OR 'hypertension'/exp OR hypertension OR 'arterial pressure'/exp OR 'arterial pressure' OR 'pulse pressure'/exp OR 'pulse pressure' OR 'glucose blood level'/exp OR 'glucose blood level' OR 'glycated hemoglobin'/exp OR 'glycated hemoglobin' OR 'hemoglobin a1c'/exp OR 'hemoglobin a1c' OR 'quantitative insulin sensitivity check index'/exp OR 'quantitative insulin sensitivity check index' OR 'insulin'/exp OR insulin OR 'homa index'/exp OR 'homa index' OR 'insulin resistance'/exp OR 'insulin resistance' OR 'c reactive protein'/exp OR 'c reactive protein' OR 'interleukin 6'/exp OR 'interleukin 6' OR 'metabolic syndrome x'/exp OR 'metabolic syndrome x' OR 'flow-mediated dilation test'/exp OR 'flow-mediated dilation test' OR 'flow mediated vasodilation'/exp OR 'flow mediated vasodilation' OR 'endothelial function'/exp OR 'endothelial function' OR 'endothelial dysfunction'/exp OR 'endothelial dysfunction' OR 'body weight'/exp OR 'body weight' OR 'body weight gain'/exp OR 'body weight gain' OR 'waist circumference'/exp OR 'waist circumference' OR 'body mass'/exp OR 'body mass' OR 'obesity'/exp OR obesity | 7,162,232 |
| #5 | #1 AND (#2 OR #3) AND #4 | 28,162,212 |
| #6 RCTs | #5 AND ('controlled clinical trial'/de OR 'randomized controlled trial'/de) | 3,000 |
| #7 Human studies | #5 AND 'human'/de AND ('controlled clinical trial'/de OR 'randomized controlled trial'/de) | 2,747 |
| #8 Adults only | #5 AND 'human'/de AND ('controlled clinical trial'/de OR 'randomized controlled trial'/de) AND ([adult]/lim OR [aged]/lim OR [very elderly]/lim) | 1,817 |
| #9 English language | #5 AND 'human'/de AND ('controlled clinical trial'/de OR 'randomized controlled trial'/de) AND ([adult]/lim OR [aged]/lim OR [very elderly]/lim) AND [english]/lim | 1798 |

**Supplemental table 4.** Syntax for searching Cochrane CENTRAL

| **ID** | **Search** | **Hits** |
| --- | --- | --- |
| #1 | MeSH descriptor: [Dairy Products] explode all trees | 5,221 |
| #2 | (dairy NEXT (product?)):ti,ab,kw (Word variations have been searched) | 1,764 |
| #3 | (milk OR cheese OR yogurt OR yoghurt OR "fermented dairy" OR "cultured dairy" OR buttermilk OR "milk powder" OR "ice cream" OR "cultured milk" OR "dairy fat" OR curd OR kefir OR butter OR cream):ti,ab,kw (Word variations have been searched) | 27,882 |
| #4 | (consumption OR intake OR consume? OR serving*):ti,ab,kw (Word variations have been searched) | 144,801 |
| #5 | #1 OR #2 OR #3 | 29,228 |
| #6 | #5 AND #4 | 8,767 |
| #7 | MeSH descriptor: [Genetic Association Studies] explode all trees | 1,131 |
| #8 | MeSH descriptor: [Polymorphism, Single Nucleotide] explode all trees | 2.534 |
| #9 | MeSH descriptor: [Genetic Variation] explode all trees | 9,104 |
| #10 | (genetic NEXT varia*):ti,ab,kw (Word variations have been searched) | 3,713 |
| #11 | (lipid NEXT polymorphism*):ti,ab,kw (Word variations have been searched) | 1 |
| #12 | (genotype OR genetic* OR allel* OR "single nucleotide polymorphism" OR "genetic risk score" OR "diet-gene interaction" OR "genetic heterogeneity" OR nutrigenetics OR nutrigenomics OR "gene-nutrient interaction" OR GWAS OR GeNuIne OR gene OR genes OR lipidom*):ti,ab,kw (Word variations have been searched) | 88,591 |
| #13 | #7 OR #8 OR #9 OR #10 OR #11 OR #12 | 88,918 |
| #14 | MeSH descriptor: [Cardiometabolic Risk Factors] explode all trees | 203 |
| #15 | MeSH descriptor: [Inflammation] explode all trees | 16,612 |
| #16 | MeSH descriptor: [Phenotype] explode all trees | 2,417 |
| #17 | MeSH descriptor: [Body Weights and Measures] explode all trees | 35,080 |
| #18 | MeSH descriptor: [Obesity] explode all trees | 21,661 |
| #19 | MeSH descriptor: [Dyslipidemias] explode all trees | 9660 |
| #20 | MeSH descriptor: [Diabetes Mellitus] explode all trees | 46,530 |
| #21 | MeSH descriptor: [Cardiovascular Diseases] explode all trees | 157,319 |
| #22 | MeSH descriptor: [Heart Disease Risk Factors] explode all trees | 672 |
| #23 | MeSH descriptor: [Stroke] explode all trees | 17,732 |
| #24 | (phenotyp* OR "body weight" OR "body mass index" OR BMI OR obesity OR obese OR overweight OR "waist circumference" OR age OR SEX OR ethnic OR race OR dyslipidemia OR diabetes OR stroke OR hypertension OR "health status" OR healthy OR premenopausal OR post-menopausal OR postmenopausal):ti,ab,kw (Word variations have been searched) | 845,099 |
| #25 | ((heart OR cardiovascular) NEXT disease*):ti,ab,kw (Word variations have been searched) | 63,363 |
| #26 | #14 OR #15 OR #16 OR #17 OR #18 OR #19 OR #20 OR #21 OR #22 OR #23 OR #24 OR #25 | 939,810 |
| #27 | MeSH descriptor: [Dyslipidemias] explode all trees | 9,660 |
| #28 | MeSH descriptor: [Cholesterol] explode all trees | 13,155 |
| #29 | MeSH descriptor: [Triglycerides] explode all trees | 8,058 |
| #30 | MeSH descriptor: [Apolipoproteins] explode all trees | 2,522 |
| #31 | MeSH descriptor: [Blood Pressure] explode all trees | 34,892 |
| #32 | MeSH descriptor: [Hypertension] explode all trees | 25,500 |
| #33 | MeSH descriptor: [Glucose Metabolism Disorders] explode all trees | 50,666 |
| #34 | MeSH descriptor: [Metabolic Syndrome] explode all trees | 3,614 |
| #35 | MeSH descriptor: [Vasodilation] explode all trees | 2,634 |
| #36 | MeSH descriptor: [Body Weight Changes] explode all trees | 12,576 |
| #37 | MeSH descriptor: [Obesity] explode all trees | 21,661 |
| #38 | MeSH descriptor: [Adiposity] explode all trees | 1,133 |
| #39 | (Cholesterol OR LDL OR HDL OR VLDL OR triglyceride* OR triacylglycerol* or dyslipidaemia* OR hyperlipidaemia OR hypertriglyceridemia* OR lipids OR lipoprotein* OR apolipoprotein* OR apoA OR apoB OR apoC OR apoD OR apoE OR apoH OR "blood pressure" OR systolic OR hyperten* OR "arterial pressure" OR "pulse pressure" OR "blood glucose" OR "plasma glucose" OR "glycated haemoglobin" OR "glycosylated haemoglobin" OR glycemi* OR HBA1C OR QUICKI OR insulin OR "homeostatic model assessment" OR HOMA* OR "insulin resistance" OR "C-reactive protein" OR interleukin OR IL6 OR "metabolic syndrome" OR "syndrome X" OR "flow-mediated dilation" OR "flow-mediated vasodilation" OR "flow-mediated vasodilatation" OR "endothelial function" OR "endothelial dysfunction" OR "body weight" OR "weight gain" OR waist OR "body mass index" OR BMI OR adiposity OR obesity):ti,ab,kw (Word variations have been searched) | 426,245 |
| #40 | #27 OR #28 OR #29 OR #30 OR #31 OR #32 OR #33 OR #34 OR #35 OR #36 OR #37 OR #38 OR #39 | 435,599 |
| #41 | MeSH descriptor: [Humans] explode all trees | 755,563 |
| #42 | MeSH descriptor: [Healthy Volunteers] explode all trees | 6,726 |
| #43 | MeSH descriptor: [Persons] explode all trees | 726,418 |
| #44 | (volunteer? OR participant* OR patient? OR subject? OR m?n OR wom?n OR individual? OR adult* OR ethnic):ti,ab,kw (Word variations have been searched) | 1,796,793 |
| #45 | #41 OR #42 OR #43 OR #44 | 1,837,935 |
| #46 | #6 AND (#13 OR #26) AND #40 AND #45 | 4,199 |
| #47 | ("randomised controlled trial" OR "randomized controlled trial" OR "randomized intervention" or "randomised int”) | 712,591 |
| #48 | #46 AND #47 | 1,687 |
|  | Results for Trials (excludes Reviews) | 1669 |
|  | Filtered for English language only | 2,916 |
